# All About the RNA: Interferon-Stimulated Genes That Interfere With Viral RNA Processes

**DOI:** 10.3389/fimmu.2020.605024

**Published:** 2020-12-09

**Authors:** Emily Yang, Melody M. H. Li

**Affiliations:** ^1^ Molecular Biology Institute, University of California, Los Angeles, Los Angeles, CA, United States; ^2^ Department of Microbiology, Immunology, and Molecular Genetics, University of California, Los Angeles, Los Angeles, CA, United States

**Keywords:** interferon-stimulated genes, co-factors, viral RNA degradation, translation inhibition, RNA sensing

## Abstract

Interferon (IFN) signaling induces the expression of a wide array of genes, collectively referred to as IFN-stimulated genes (ISGs) that generally function to inhibit viral replication. RNA viruses are frequently targeted by ISGs through recognition of viral replicative intermediates and molecular features associated with viral genomes, or the lack of molecular features associated with host mRNAs. The ISGs reviewed here primarily inhibit viral replication in an RNA-centric manner, working to sense, degrade, or repress expression of viral RNA. This review focuses on dissecting how these ISGs exhibit multiple antiviral mechanisms, often through use of varied co-factors, highlighting the complexity of the type I IFN response. Specifically, these ISGs can mediate antiviral effects through viral RNA degradation, viral translation inhibition, or both. While the OAS/RNase L pathway globally degrades RNA and arrests translation, ISG20 and ZAP employ targeted RNA degradation and translation inhibition to block viral replication. Meanwhile, SHFL targets translation by inhibiting -1 ribosomal frameshifting, which is required by many RNA viruses. Finally, a number of E3 ligases inhibit viral transcription, an attractive antiviral target during the lifecycle of negative-sense RNA viruses which must transcribe their genome prior to translation. Through this review, we aim to provide an updated perspective on how these ISGs work together to form a complex network of antiviral arsenals targeting viral RNA processes.

## Introduction

Organisms must constantly defend themselves against viral pathogens. In order to stem viral spread, cells must both signal the presence of viral infection and hinder their replication. One key first line of cellular defense in vertebrates is the type I interferon (IFN) response. Hosts possess sensors which recognize pathogen-associated molecular patterns (PAMPs) of invading viruses such as the viral replicative intermediate double-stranded RNA (dsRNA) and activate transcription factors such as IFN-regulatory factors 3 or 7 (IRF3/7) and nuclear factor kappa-light-chain-enhancer of activated B cells (NFκB). As a result, these transcription factors translocate to the nucleus to activate expression of type I IFN and other proinflammatory cytokines.

The type I IFN receptor is expressed ubiquitously on almost all cell types, allowing for IFN signaling in both infected and neighboring cells that are uninfected. Janus kinase-signal transducer and activator of transcription (JAK-STAT) is the predominant, canonical pathway that regulates ISG transcription. IFN binding to its cell surface receptor, comprised of IFN-α receptor 1 (IFNAR1) and IFN-α receptor 2 (IFNAR2), leads to phosphorylation of pre-associated JAK1. Phosphorylated JAK1 and tyrosine kinase 2 (TYK2) then phosphorylate the IFN receptor, which recruits STAT1/2 to be phosphorylated themselves. Phosphorylated STAT1/2 recruit IRF9 to form the transcription factor complex IFN-stimulated gene factor 3 (ISGF3). ISGF3 translocates to the nucleus where STAT1 is further phosphorylated for full activation. Within the nucleus, ISGF3 binds to IFN-stimulated response elements present in the promoters of IFN-stimulated genes (ISGs), which then effect an antiviral cellular environment [for a comprehensive review on IFN signaling, see (1)].

Interestingly, a growing body of evidence in recent years suggests a plethora of non-canonical mechanisms [for a comprehensive review on canonical and non-canonical regulation of ISG transcription, see (2)]. Non-canonical ISGF3 complexes containing unphosphorylated STAT2, unphosphorylated STAT1 and STAT2, or STAT2 and IRF9 only have been found to mediate expression of specific ISGs (3–6). Other transcription complexes such as STAT5-CrkL (7, 8) or transcription factors such as IRF1 (9) can induce ISG expression. Additionally, cytokines such as TNF-α can moderately induce a subset of ISGs through the NFκB protein complex and further synergize with type I or II IFN to jointly upregulate antiviral ISG expression (10–13). Surprisingly, cellular pathways that have no apparent connection to the innate immune response have been linked to ISG induction. For example, inhibitors of nucleotide synthesis have been shown to effectively upregulate ISG expression in a JAK-STAT-independent manner (14–17). Differences in the extent and timing of ISG upregulation likely depend on the complex interplay between these various mechanisms.

**Figure 1:**
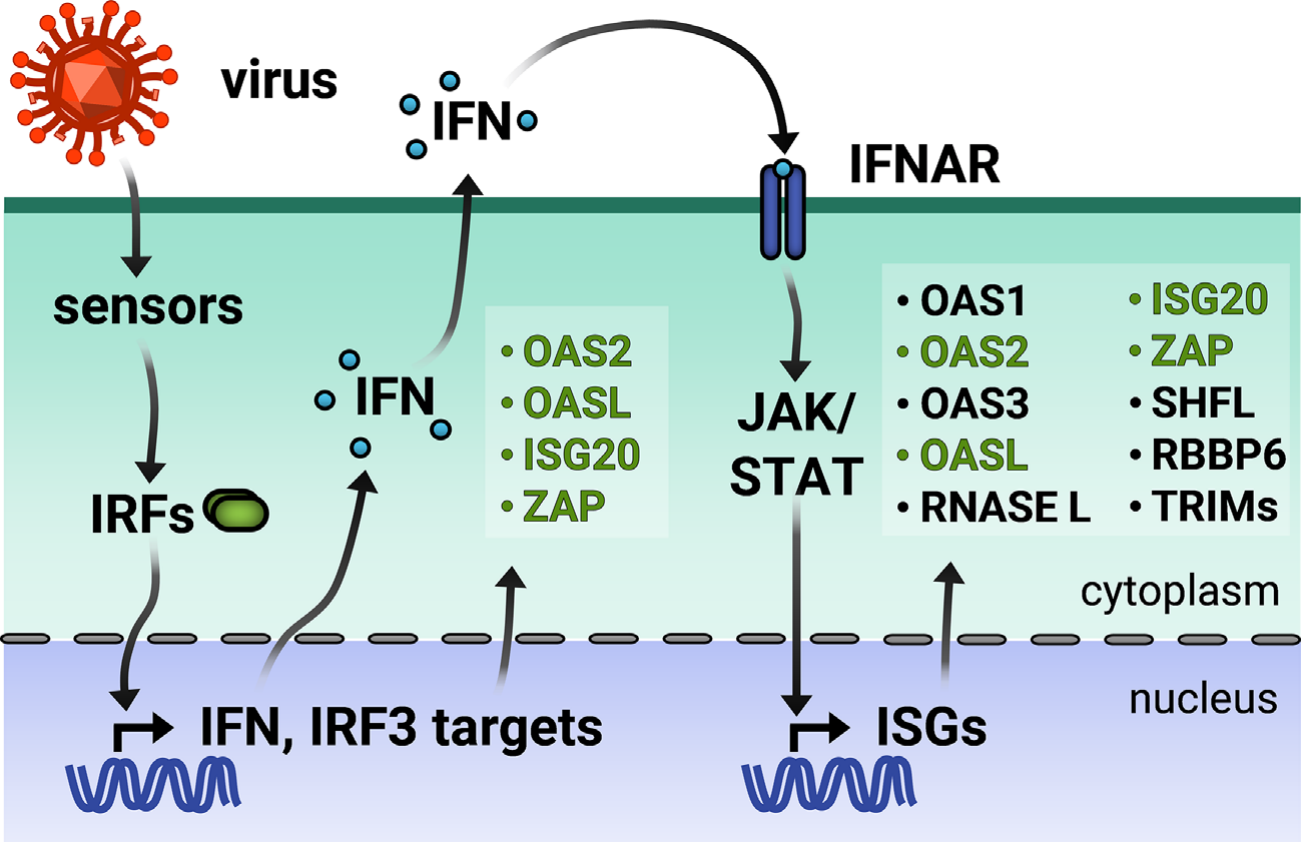
Viral infection triggers the type I interferon (IFN) response. Virus entry triggers cellular pathogen sensors, which induce production of IFN and IFN regulatory factor 3 (IRF)-dependent genes. Type I IFN is secreted and binds to IFN-α receptor on the same and neighboring cells, activating production of a wider array of IFN-stimulated genes (ISGs). IRF3-dependent ISGs are colored in green. TRIMs is not inclusive of the entire TRIM family, but only those inducible by IFN.

Broadly speaking, an ISG is any gene whose expression is induced by IFN signaling. Advances in RNA-sequencing (RNA-seq) technology have enabled the identification of ISGs across varied cell lines by measuring changes in the transcriptome in response to IFN stimulation. The online database INTERFEROME continues to catalog the results of such gene profiling studies (18). However, ISG expression is more nuanced in reality. A subset of ISGs are direct targets of IRF3/7 and can be induced with or without downstream IFN signaling (**Figure 1**) (1). Other ISGs are both basally expressed and IFN-inducible, while still others are cell-type specific (19, 20). Moreover, there are three types of IFN, wherein type I and III are the classic antiviral IFNs. Though type I and III IFNs bind to different receptors, they signal through the same JAK-STAT pathway, thus inducing a shared array of ISGs. Still, type I and III IFN signaling pathways are differentiated by expression kinetics and cell-type specific receptor expression [for a recent review, see (21)]. Tight regulation of ISG expression is necessary because dysregulation of the type I IFN response results in interferonopathies or systemic inflammation deleterious to the organism (22).

In addition to regulating their own expression, ISGs are well known for their inhibition of viral replication. They employ diverse mechanisms to block virtually every step of viral replication, though ISGs have been shown to target different viral life cycle stages for different viruses [for recent reviews on a broad range of antiviral ISGs, see (20, 23)]. For example, the IFITM family blocks viral entry of diverse viruses (24) while the Mx GTPases recognize diverse nucleocapsids and block their nuclear import (25). TRIM5α disrupts retrovirus uncoating and targets several viral proteins for proteasomal degradation (26). However, these only represent a tip of the iceberg. Recent advances in systematic approaches have allowed us to unbiasedly uncover ISGs with previously uncharacterized antiviral activity. Compiled ISG libraries have facilitated focused loss-of-function or gain-of-function screens of hundreds of ISGs, illuminating the contribution of individual ISGs in varied viral contexts (27–30). Furthermore, as advances in omics approaches allow examination of cellular changes on a systemic level, attention is shifting to how ISGs interact and even synergize with one another (27, 31, 32). Moreover, detailed mechanistic studies are still needed in order to unravel their mode of action. As ISGs may employ different antiviral mechanisms against different viruses, studies in varied viral systems will illuminate how ISGs might recruit different cellular pathways or factors.

**Figure 2:**
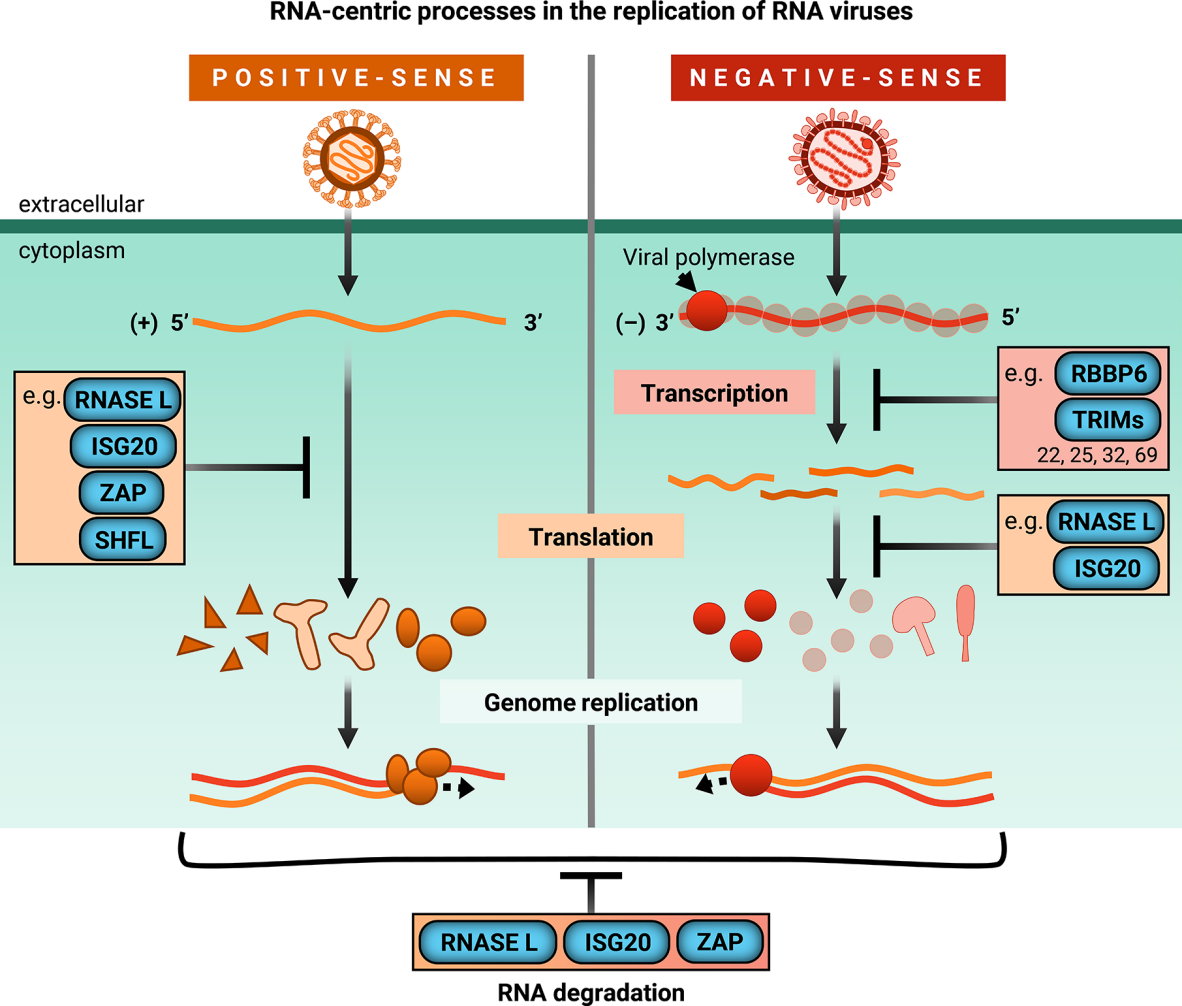
ISGs inhibit RNA-centric processes in replication of RNA viruses. Upon infection, positive-sense single-stranded RNA genomes are directly translated by host machinery. Following expression of viral replicase proteins, genome replication occurs. However, negative-sense single-stranded RNA viruses bring their own RNA-dependent RNA polymerase and viral co-factors to transcribe their genomes before the viral mRNAs can be translated by host machinery for genome replication. ISGs covered in this review are shown to block global or viral translation (RNaseL, ISG20, ZAP, SHFL) and viral transcription (RBBP6, TRIMs). They can also inhibit viral replication by degrading cellular and/or viral RNA (RNase L, ISG20, ZAP). TRIMs that inhibit transcription are limited to those mentioned in this review, specifically TRIM22, TRIM25, TRIM32, and TRIM69.

Rather than provide a comprehensive, surface-level view of myriad ISGs with their arrayed antiviral functions, we have chosen to focus on a subset of ISGs that interfere with viral RNA processes. Recent studies have provided an emerging view on the diversity and complexity of RNA-based mechanisms by which different ISGs inhibit viruses with an RNA genome. With the exception of retroviruses which replicate through a DNA intermediate, single-stranded RNA (ssRNA) viruses can generally be categorized as positive-sense and negative-sense. Positive-sense (+) ssRNA viruses possess genomes that generally resemble mRNA, in that it can be translated directly by host translation machinery. However, negative-sense (-) ssRNA viruses code their proteins in the reverse orientation. Therefore, they must package their own RNA-dependent RNA polymerases and transcribe their genomes before viral protein synthesis can occur. While there are many more ISGs that inhibit viral RNA processes (**Tables 1** and **2**), this review will highlight recent exciting work on a subset of ISGs that act in an RNA-centric manner to sense, degrade, or inhibit transcription or translation of both (+) and (-) ssRNA viral genomes (**Figure 2**). We have chosen the ISGs here because at the time of writing of this review, they have not been comprehensively reviewed in the antiviral innate immunity field, and exciting developments have either illuminated nuances of well-characterized mechanisms or uncovered entirely new mechanisms by which these ISGs inhibit viral replication. We synthesized diverse antiviral mechanisms of individual ISGs and provided hypotheses for how cellular co-factors mediate the distinct antiviral activities of these ISGs, which not many previously published reviews have done. We will begin our discussion with the 2’-5’ oligoadenylate synthetase (OAS)/RNase L pathway, which both senses and degrades RNA, making it a potent early inhibitor of viral replication. We will then segue into ISGs that both degrade RNA and inhibit translation, such as ISG20 and zinc finger antiviral protein (ZAP), before focusing on a novel means of translation inhibition by Shiftless (SHFL). We will end with a discussion of E3 ligases that inhibit viral transcription. Though there are many additional ISGs that block these RNA-centric steps of viral replication, this review focuses on ISGs that possess multiple or seemingly contradictory antiviral mechanisms. Protein-protein interactions or cellular co-factors could explain diverse antiviral mechanisms of individual ISGs.

**Table 1:** ISGs that must bind RNA to inhibit viral RNA processes.

Gene	Mode of inhibition	RNA motif	References
ADAR1	A-to-I sequence conversion	dsRNA	(33)
IFIT1, -2, -3, -5	Translation inhibition	Type 0 cap structure lacking 2’-*O*-methylation at the 5’ end of ssRNA	(34, 35)
ISG20	RNA degradation, translation inhibition	Largely unknown; recognizes m^6^A on HBV RNA	(36–38)
MDA5	RNA sensor	Long cytosolic dsRNA (> 1,000-2,000 bp)	(39–41)
OAS1, -2, -3	RNA sensor, synthesize 2-5A to activate RNase L	dsRNA	(40, 42)
OASL	RNA sensor, promotes RIG-I signaling and inhibits cGAS signaling	dsRNA	(43)
PARP12	Translation inhibition	Unknown	(44–46)
PKR	RNA sensor, translation inhibition	dsRNA	(40)
RIG-I	RNA sensor	short cytosolic dsRNA or ssRNA (10-300 bp) with 5’-triphosphate ends, enriched in poly-U/UC or AU sequences	(39–41)
RNase L	RNA degradation	ssRNA, cleaves at ^ in U^N, where N is any nucleotide	(47)
TRIM25	Translation inhibition	Unknown	(48–50),
ZAP	RNA degradation, translation inhibition, ISG synergy	ssRNA, CG dinucleotide	(23)
ZCCHC3	RNA sensor	dsRNA	(51)
ZNFX1	RNA sensor	dsRNA	(52)

Mode of inhibition, RNA motif or substrate preference, and references for the most recent reviews are listed for ISGs that bind RNA to inhibit viral RNA processes.

**Table 2:** ISGs that inhibit viral RNA processes with no known dependence on RNA binding.

Gene	Inhibited process	Known mechanism(s)	References
RBBP6	Transcription	Competitively binds to viral RNA polymerase	(53)
SHFL	Translation	Inhibits -1 ribosomal frameshifting	(54)
TRIM22	Transcription	Prevents transcription factor binding to HIV-1 promoter	(55)
TRIM25	Transcription	Blocks IAV RNA elongation	(56)
TRIM32	Transcription	Targets IAV polymerase for degradation	(57)
TRIM69	Transcription	Sequesters VSV_IND_ P	(58, 59)
Viperin	Viral RNA synthesis	Synthesizes chain terminator from cytidine triphosphate	(60)

Viral RNA process inhibited (viral RNA synthesis, transcription, or translation) known mechanism(s), and references for the most recent reviews are listed for ISGs that inhibit viral RNA processes without a strict requirement for RNA binding.

## OAS/RNase L: Sensing Viral PAMP Triggers Global RNA Degradation and Translational Arrest

Degrading viral genomes presents one potent method of antiviral activity; digesting viral genetic material ensures that no further steps in replication can occur. However, the challenge lies within being able to control RNA degradation to ensure cellular survival or limit destruction within the host. The OAS/RNase L pathway is activated upon sensing the PAMP of dsRNA, serving two functions: sensing viral intruders and inhibiting viral replication by degrading RNA almost indiscriminately, inducing global translational arrest.

**Figure 3:**
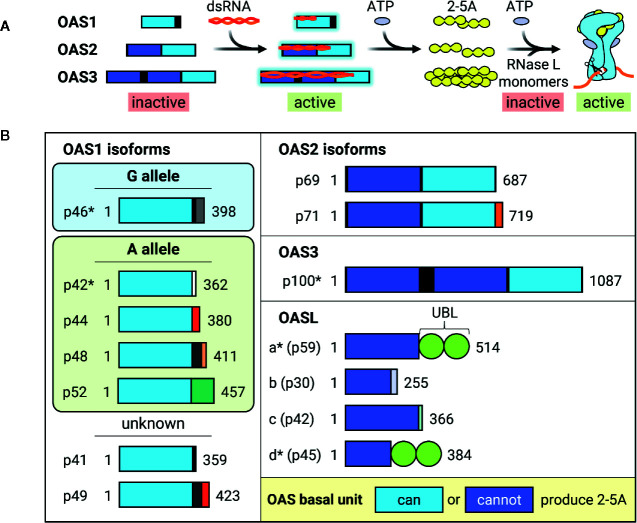
Activation and dsRNA binding of OAS isoforms. **(A)** Diagram of how OAS is activated by viral dsRNA and synthesizes 2-5A to activate RNase L. Longer OAS isoforms are activated by longer dsRNA. Though all are able to synthesize 2-5A, OAS3 2-5A synthesis is necessary and sufficient for RNase L activation. RNase L exists as inactive free monomers, but dimerizes and activates upon binding to 2-5A and ATP, thereby cleaving RNA. Interestingly, IFN and some ISG transcripts are resistant to RNase L cleavage, resulting in preservation of antiviral signaling. **(B)** Schematic of known human OAS isoforms encoded by OAS genes OAS1, OAS2, OAS3, and OASL. Isoforms marked with * have demonstrated direct antiviral activity. Length of boxes approximates gene length, with different colored C-terminal boxes representing different coding sequences. Light blue OAS basal units are able to synthesize 2-5A, while dark blue OAS basal units are catalytically inactive. Most OAS1 isoforms are expressed by either G or A alleles, which differ by the inclusion of a G or A nucleotide at a splice acceptor site. However, OAS1 p41 and p49 have not been attributed to either G or A and are therefore labelled as “unknown.” UBL, Ubiquitin-like domain.

The OAS/RNase L pathway was one of the first ISG antiviral mechanisms to be identified and elucidated in the 1970s [reviewed in (61–64)]. Binding to dsRNA activates OAS, which then synthesizes 2’-5’ oligoadenylates (commonly abbreviated as 2-5A) that in turn activate RNase L to cleave cytoplasmic RNA, thereby inhibiting viral replication (**Figure 3A**). Humans possess three catalytically active OAS genes (OAS1-3) and one inactive gene (OASL). Each OAS is composed of 1 (OAS1 and OASL), 2 (OAS2), or 3 (OAS3) basal OAS units (**Figure 3A**). While only the C-terminal OAS unit in each protein is catalytically active and responsible for synthesizing 2-5A, both active and inactive OAS units can still bind dsRNA. RNase L, present in cellular cytoplasm as an inactive monomer, forms a catalytically active dimer upon binding to 2-5A and cleaves RNA in a seemingly indiscriminate manner. Though OAS1-3 all carry signatures of positive selection, which is indicative of rapid evolution resulting from host-pathogen interactions, OASL does not. Furthermore, OAS1 displays much stronger signatures than OAS2 or OAS3 (65, 66). RNase L also carries signatures of positive selection and some of the positively selected residues are located within the RNA-binding domain (66).

In the past 10 years, great strides have been made in clarifying dsRNA substrate specificity and 2-5A synthesis activity of individual OAS isoforms due to the advent of CRISPR-Cas9 gene-editing techniques and generation of OAS knockout cell lines and mouse models. Moreover, sweeping improvements in genome-wide RNA-seq and in-depth proteomics have illuminated new intricacies of OAS/RNase L-mediated inhibition of viral replication, which we will review here.

### OAS1-3: 2-5A Messenger Synthesis

Recent years have not only seen a flurry of biochemical and structural studies on individual OAS paralogs, which have advanced understanding of their RNA substrate specificity and activation, but also of their individual splice variants. Surprisingly, OAS3 has been identified as the main contributor to the OAS/RNase L pathway, as OAS3-mediated 2-5A synthesis has been demonstrated as necessary and sufficient for RNase L pathway (67).

Though there are four OAS genes in humans, alternative splicing of additional 3’ exons generates 10 different catalytically active and 4 inactive isoforms (**Figure 3B**) (62, 68, 69). OAS isoforms can vary greatly in size and degree of catalytic activity within the same gene (69). Though OAS1 isoforms differ from each other in their C-terminal regions, all are able to synthesize 2-5A *in vitro* after incubation with poly(I:C), a dsRNA mimetic, with isoforms p42 and p46 expressed more highly in HEK293 cells (69). A yeast two-hybrid screen for p42 and p44, predicted to be expressed by all humans (70), revealed different binding partners, suggesting that OAS1 unique C-terminal regions may alter protein-protein interactions mediating isoform-specific functions (69). In support of this hypothesis, p46 possesses a CaaX prenylation motif that causes it to localize to mitochondria, whereas p42 lacks the CaaX motif and is cytoplasmic (71, 72). This difference in cellular localization is thought to contribute to their divergent impact on cellular respiration upon overexpression (72), and could contribute to their differential antiviral activity.

Genome-wide association studies of 2-5A synthesis activity revealed the presence of two OAS1 alleles which differ by the presence of a G or A at a splice acceptor site (70). Individuals with the G allele have high 2-5A activity and express the OAS1 p46 isoform, whereas individuals with 1-2 copies of the A allele have significantly lower OAS activity and express the OAS1 p42, p44, p48, and p52 isoforms (**Figure 3B**) (70, 73). This G/A single nucleotide polymorphism is associated with a variety of infectious diseases, such as the (+) ssRNA viruses West Nile virus (WNV) and hepatitis C virus (HCV), in addition to a hepatitis B virus (HBV)-associated autoimmune disease; wherein the G allele confers resistance and the A allele is associated with higher risk (74–78). A comprehensive study of OAS isoform-specific antiviral activity against dengue virus (DENV) found that out of all catalytically active isoforms, only OAS1 p42 and p46 and OAS3 p100 were able to block DENV replication through an RNase L-dependent mechanism (79). Furthermore, degree of antiviral activity was positively correlated with amount of RNase L activity, as measured by 28S and 18S rRNA cleavage, wherein OAS3 inhibited DENV replication more robustly than either OAS1 isoform.

This finding of RNase L-dependent antiviral activity of OAS3 was surprising at the time. OAS3 was thought to primarily synthesize the minimal dimeric 2-5A molecule, composed solely of two adenylate groups, which poorly activates RNase L (80, 81). More recently, it was shown that not only does OAS3 require 3-4 logs lower concentration of dsRNA than OAS1 for 2-5A synthesis, but it also readily synthesizes 2-5As of 3 or more linked ATPs both *in vivo* and *in vitro* (82). Increased OAS3 sensitivity to dsRNA can be explained by its additional, catalytically inactive OAS units, which retain ability to bind dsRNA (83). Though catalytically inactive itself, deletion of the most N-terminal OAS unit in OAS3 (OAS3.DI) nearly abolished OAS3 catalytic activity and dsRNA binding (83). Given that OAS3 is thought to adopt an elongated conformation (82), dsRNA binding by OAS3.DI may determine OAS3 preference for longer dsRNA substrates, and may explain its greater dependency on dsRNA length for activation as compared to OAS1 (83, 84). This could help OAS3 discriminate self from non-self dsRNA, as long dsRNA is absent from uninfected cells (85). These studies culminated with the novel finding that OAS3 is the primary driver of RNase L antiviral activity (67). The replication of both DNA (vaccinia virus (VV)) and RNA viruses ((+) ssRNA: WNV, Sindbis virus (SINV); (-) ssRNA: influenza A virus (IAV)) was tested in OAS1-3 single KO cells. While wild-type WNV and SINV trigger the OAS/RNase L pathway, wild-type VV and IAV do not due to their active antagonism of the pathway. Therefore, mutant VV and IAV strains that lacked viral antagonists were used. OAS3 KO cells have negligible rRNA degradation and 2-5A production during both poly(I:C) treatment and viral infection, in stark contrast to OAS1 and OAS2 KO cells which had high rRNA degradation and 2-5A production (67). This generally correlated with inhibition of virion production, as OAS3 and RNase L single KO cells had significantly higher viral titers than parental and single OAS1 and OAS2 KO cells for all viruses tested. These data suggest that OAS3 is both necessary and sufficient to drive RNase L-dependent antiviral activity against diverse viruses.

Though OAS3 possesses a dominant role in RNase L-dependent antiviral activity, more antiviral and cellular roles of OAS1 and OAS2 are beginning to be elucidated. In fact, OAS1 and OAS2 are responsible for some antiviral activity against WNV and SINV, albeit to a lesser extent than OAS3/RNase L (67). OAS2 was found to inhibit the (+) ssRNA virus Zika virus (ZIKV) replication through positive regulation of IFN signaling (86). OAS2 may also play a role in lactation, as it was identified in a screen for genes with roles in mammary development (87). Moreover, a novel role for OAS1 and 2-5A synthesis was recently identified in the context of poly-ADP-ribosylation (PAR) and DNA damage-induced cell death (88). As part of the DNA damage response, poly-ADP-ribose polymerase 1 (PARP1) synthesizes PAR polymers to recruit and modify DNA repair proteins (89). Upon resolution of the response, PAR products are hydrolyzed and can trigger apoptosis. ADP-ribose is a known substrate of OAS (90) and addition of 2-5A linkage onto ADP-ribose effectively functions as a chain terminator of PARylation (88). As a result, OAS1 p42 protects cells from DNA damage-induced death by reducing PARP1-mediated PARylation. The absence of OAS1 dramatically increased PAR accumulation within cells upon H_2_O_2_ treatment, which was not rescued by a catalytically inactive OAS1 mutant (88). These data agree with the observation that cancer cells resistant to DNA-damaging therapies frequently highly express OAS1 (91, 92), as it is hypothesized OAS1 confers resistance to DNA damage-induced cell death.

### RNase L: RNA Degradation and Global Translational Arrest

Upon activation by 2-5A, ubiquitous RNA degradation by RNase L simultaneously inhibits many facets of viral replication. Not only does RNase L activity directly degrade single-stranded cellular and viral RNAs, but it also promotes apoptosis, stimulates immune signaling, and induces rapid translational arrest (61).

Activation of RNase L quickly arrests global translation. This rapid translational arrest is traditionally attributed to degradation of transcripts involved in host translation machinery, as evidenced by degradation of 28S and 18S rRNA upon RNase L activation. However, closer examination revealed that at least 50% of 28S rRNA remains and overall tRNA levels are unchanged at the onset of global translation shut-off, challenging this prevailing hypothesis (93). Thus, degradation of translational machinery transcripts cannot explain early translational arrest (93). Another group presents the compelling hypothesis that translational arrest results from almost indiscriminate degradation of cellular RNA by RNase L, crippling gene expression by depriving ribosomes of substrates (94). Comparison of mRNA abundance in parental and RNase L KO A549s revealed reduction in almost all abundant mRNAs in parental, but not in RNase L KO cells after poly(I:C) stimulation. However, some mRNAs are resistant to RNase L cleavage, such as *IFN-β*. Furthermore, mRNAs that substantially increase in both parental and RNase L KO cells in response to poly(I:C) are highly enriched for ISGs such as *IFIT2*, *OAS2*, *MDA5*, and *RIG-I*, suggesting that antiviral mRNAs are resistant to RNase L turnover (94). Determinants of RNase L resistance have yet to be identified. These data are compatible with the independent observation that both type I and III IFN are still produced from seemingly translationally arrested cells (95). Together, these data support a model in which the rapid translational arrest by the OAS-RNase L pathway is doubly beneficial to the organism by both inhibiting viral replication and by permitting antiviral signaling to inhibit viral spread within the host.

RNase L structural studies have uncovered nuances of RNase L substrate selectivity. The first near full-length human RNase L crystal structure allowed for detailed analysis of RNase L dimerization, substrate recognition, and ribonuclease activity (96). In agreement with previous studies that found RNase L cleavage after UU and UA dinucleotides (97), recent structural analysis suggests that RNase L recognizes and cleaves the pattern UN^N (N: any nucleotide, ^: cleavage site) (96). Two different RNA-seq approaches have been utilized to identify RNase L substrates. One approach sequenced RNAs enriched for poly-A-tails after incubating lysates with either 2-5A or pre-activated RNase L. Their results suggest that RNase L selectively degrades transcripts similar to those regulated by miRNAs, achieving a redundant outcome of suppression of mammalian cell adhesion and proliferation (98, 99). In contrast, another approach capitalized on the characteristic 2’,3’-cyclic phosphate termini of RNase L cleavage products. They used a 2’,3’-cyclic phosphate RNA-seq analysis in order to identify small RNA cleavage products (93). Here, it was found that most highly-upregulated reads map to tRNAs and Y-RNAs. Analysis of these RNAs revealed site-specific cleavage in both tRNAs and Y-RNAs, which may be shaped by post-transcriptional modifications. Interestingly, though cleavage sites of the tRNAs and other Y-RNAs followed UN^N specificity, the Y-RNA RNY4 was cleaved at CA^G. This unusual cleavage was not recapitulated in the absence of cellular proteins, suggesting that RNase L may acquire site-specificity by recognizing protein/Y-RNA complexes. In this way, RNase L could require co-factors to determine cleavage specificity. It is interesting to speculate that putative co-factors bound to ISG transcripts could also shield cleavage sites to enable their escape from RNase L recognition.

### OASL

Though OASL lacks the ability to synthesize 2-5A and thus to participate in RNase L-dependent antiviral activity, it is still potently induced upon IFN stimulation and is also a direct target of IRF3 (100). OASL is composed of an N-terminal, catalytically inactive basal OAS unit followed by a tandem ubiquitin-like domain (UBL) (**Figure 3B**) (43) and possesses antiviral activity against several RNA viruses (27, 68, 101, 102). However, OASL promotes replication of the DNA virus, Kaposi sarcoma herpesvirus (KSHV) (103). These conflicting data can be reconciled by the recently uncovered divergent roles of OASL in both enhancing signaling of the dsRNA sensor RIG-I, thus inhibiting replication of RNA viruses (104), and inhibiting signaling of the cytoplasmic dsDNA sensor cGAS, hence promoting replication of DNA viruses (105, 106). Activation of RIG-I requires its simultaneous binding to dsRNA and poly-ubiquitin chains (107). OASL was shown to interact and colocalize with RIG-I, enhancing RIG-I signaling *via* its C-terminal UBL domain which acts as a poly-ubiquitin mimic to activate RIG-I (104). Furthermore, OASL antiviral activity is suggested to be completely RIG-I dependent, as its viral inhibition is abolished in the absence of RIG-I (104).

Meanwhile, two independent groups simultaneously identified the role of OASL in suppressing cGAS activity (105, 106). One group took a proteomics-based approach to uncover cGAS interactors in the context of herpesvirus infection, identifying OASL as a cGAS interactor and inhibitor (105). Another group utilized a targeted approach to assess the role of OASL during infection with varied DNA viruses, and verified the importance of OASL in promoting DNA viral replication *in vivo* by using a murine model for OASL KO (106). They observed that OASL deficiency results in increased IFN induction and reduced viral titers (106). Both groups found that the OAS-like domain is responsible for interacting with cGAS and that OASL-cGAS interaction is independent of cGAS DNA binding (105, 106). Enzyme inhibition kinetics experiments with OASL, cGAS, and the cGAS substrates ATP and GTP showed that OASL non-competitively inhibits cGAS production of its signaling molecule, cGAMP (106).

The OAS/RNase L pathway effectively inhibits viral replication by linking viral sensing to global inhibition of cellular processes. Widespread RNA degradation by activated RNase L globally not only arrests translation, preventing viruses from synthesizing new proteins, but also still allows for IFNs and several ISGs to be translated, promoting the establishment of an antiviral environment. Furthermore, the catalytically inactive OASL functions as a double-edged sword in its modulation of innate immune signaling, simultaneously inhibiting replication of RNA viruses while enhancing replication of DNA viruses.

## Multifaceted RNA-Dependent Antiviral Mechanisms: From Targeted Viral RNA Degradation to Translation Inhibition

The following three ISGs are like the OAS/RNase L pathway in inhibiting viral translation, but dissimilar in their mechanism of inhibition. While the OAS/RNase L pathway employs global translation inhibition, ISG20, ZAP, and SHFL target specific RNA substrates. Furthermore, both ISG20 and ZAP have been shown to directly or indirectly degrade viral RNA, but their RNA degradation differs from OAS/RNase L in two major ways. They target specific viral RNA substrates for degradation, and this activity is independent of their inhibition of viral translation. The multiple, diverse, independent antiviral mechanisms of ISG20, ZAP, and SHFL can be explained through their recruitment of different co-factors, which this section will explore in depth.

### ISG20

Antiviral activity of ISG20 has been attributed to two distinct mechanisms so far: degradation of viral RNA and translation inhibition, which can be direct or indirect. ISG20 was first identified as upregulated in response to IFN over 20 years ago (108). ISG20 is expressed in both the cell nucleus and cytoplasm, and is part of the DEDDh subgroup of the larger 3’ to 5’ DEDD exonuclease superfamily, which possesses a large exonuclease domain (EXO III domain) of about 150 amino acids that can confer DNase and/or RNase activity. The EXO III domain is characterized by three distinct exonuclease motifs defined by four invariant amino acids which lend this superfamily its name: three aspartate (D) and one glutamate (E) residue (109). The DEDDh subgroup also includes a conserved histidine residue. DEDDh exonucleases share a conserved fold and active site but have divergent substrate-binding sites, allowing them to recognize and thus degrade different substrates. Biochemical studies have shown that ISG20 degrades both ssRNA and DNA, with higher nuclease activity against RNA substrates (110).

Because ISG20 is a 3’ to 5’ RNA exonuclease, it has long been thought that ISG20 antiviral activity results primarily from degradation of viral RNA. In line with this hypothesis, ISG20 has been shown to exhibit antiviral activity primarily against RNA viruses (111). Overexpression and knockdown experiments show that ISG20 widely suppresses viral replication of diverse (+) ssRNA viruses from *Togaviridae* (SINV), *Flaviviridae* (yellow fever virus (YFV), WNV, HCV), *Picornaviridae* (encephalomyocarditis virus, hepatitis A virus), and (-) ssRNA viruses from *Rhabdoviridae* (vesicular stomatitis virus (VSV)), *Orthomyxoviridae* (IAV), and *Bunyaviridae* (111, 112). ISG20 also inhibits human immunodeficiency virus-1 (HIV-1) and HBV, a DNA virus with an RNA replicative intermediate (36, 37, 113–115). However, ISG20 does not display pan-antiviral activity, as it fails to inhibit severe acute respiratory syndrome coronavirus (SARS-CoV), a member of the (+) ssRNA virus family *Coronaviridae* (38), and adenovirus, a DNA virus (111). The hypothesis that ISG20 degrades viral RNA is supported by reduced expression of various viral mRNAs and replicons in the presence of catalytically active, but not catalytically inactive ISG20 (36, 111, 112, 116).

**Figure 4:**
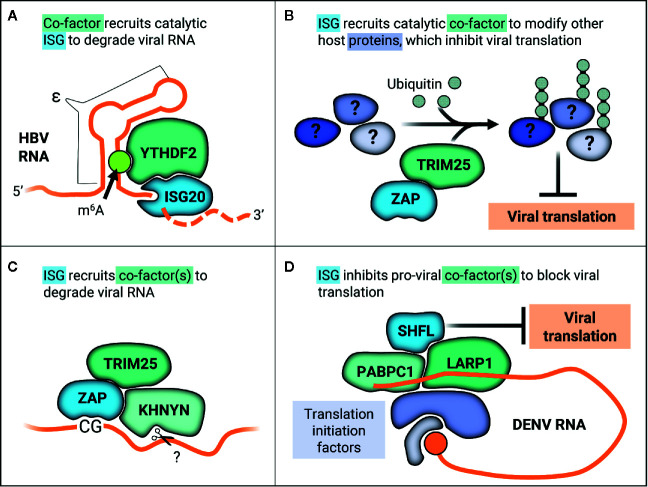
ISGs work with co-factors in varied ways to inhibit viral replication through diverse mechanisms. **(A)** YTHDF2 recognizes m^6^A methylation on the ϵ in HBV RNA transcripts, recruiting catalytic ISG20 to degrade HBV RNA. **(B)** ZAP recruits TRIM25 to ubiquitinate other host proteins, which may alter their native cellular or viral-associated activities in order to inhibit viral translation. **(C)** ZAP recognizes CG motifs in viral RNA and forms a complex with TRIM25 and the endonuclease KHNYN, which then putatively cleaves ZAP-bound RNA. **(D)** SHFL inhibits DENV replication by interacting with the viral RNA 3’UTR binding proteins PABPC1 and LARP1 and potentially inhibiting recruitment of further translation machinery.

One long-standing question has been how ISG20 selectively degrades RNA, given that ISG20 does not have any apparent regulatory domains and that its overexpression does not decimate cellular RNA. It is thought that ISG20 may interact with cellular co-factors, supported by its cell-type specific inhibition of YFV (38). The cellular N^6^-methyladenosine (m^6^A) pathway has been linked to ISG20 antiviral activity in the context of HBV infection. Briefly, m^6^A is the most common reversible post-transcriptional modification that occurs on cellular RNAs. The m^6^A pathway involves three primary types of proteins—writers, erasers, and readers—which respectively add, remove, and bind m^6^A (117). Readers affect stability, translation, and localization of m^6^A mRNA (117). One main group of m^6^A readers is the YT521-B homology (YTH) domain-containing proteins. The cytoplasmic YTH members (YTHDF1-3) were recently found to play redundant roles in mediating degradation of m^6^A-mRNAs (118). HBV transcripts are methylated within the epsilon stem-loop structure (ϵ), which is present at the 3’ end of all HBV RNAs and repeated twice in the pregenomic RNA (pgRNA) (37). The HBV polymerase binds the 5’ ϵ in pgRNA to initiate packaging and reverse transcription. ISG20 was shown to inhibit HBV replication by binding to ϵ (115). Furthermore, ϵ contains a conserved m^6^A consensus sequence that negatively regulates HBV RNA stability in a YTHDF2-dependent manner (119). It was then demonstrated that YTHDF2 and ISG20 interact in an HBV-independent manner and that depletion of YTHDF2 abolishes IFN-dependent HBV RNA degradation (37). They propose that m^6^A modification of ϵ is recognized by YTHDF2, which then recruits ISG20 to target HBV pgRNA for RNA degradation (**Figure 4A**). Based on these data, YTHDF2 was identified as an essential ISG20 co-factor, marking the first time any group has identified a regulator of ISG20 substrate specificity.

Still, the role of RNA degradation in ISG20 antiviral activity remains open for debate. Multiple studies have shown that ISG20 overexpression inhibits viral replication in an exonuclease-dependent manner, and several have observed a corresponding decrease in viral RNA (38, 111–113, 120). Moreover, most studies on ISG20 have utilized the catalytically inactive mutant D94G (110) to support the hypothesis that ISG20 exonuclease activity is required for antiviral activity (38, 111–113, 116, 120, 121). This invariant residue is crucial to ISG20 structure as it helps coordinate an Mn^2+^ ion in the active site (122). It is unknown what other deleterious effects D94G may have on the overall structure of ISG20. One group mutated all Asp residues in the DEDDh catalytic motif to Gly (D11G, D94G, and D154G) and found that while all three mutations abolished exonuclease activity, only D11G and D94G also lost antiviral activity against HBV. Surprisingly, the exonuclease-deficient ISG20-D154G was still able to inhibit HBV replication (36). This suggests that ISG20 possesses both exonuclease-dependent and -independent antiviral activities.

Recently, two groups have independently found that ISG20 mediates antiviral activity through translation inhibition and not RNA degradation, but propose two divergent mechanisms (121, 123). One group proposes that ISG20-mediated translation inhibition is indirect, mediated through ISG20-dependent upregulation of other ISGs (123). They found that overexpression of ISG20 upregulates expression of many IRF3-dependent genes in mouse embryonic fibroblasts, such as IFIT1, an ISG that inhibits translation of viral RNA with non-2’O-methylated 5’ caps (124). Furthermore, they showed that mutant alphavirus normally recognized and attenuated by IFIT1 is equally virulent as the wild-type virus in the absence of ISG20 *in vivo*, suggesting that ISG20 inhibits translation by promoting IFIT1 expression (123). On the contrary, the second group proposes that ISG20 is critical for discrimination of non-self nucleic acids from self to inhibit viral translation (121). By examining luciferase reporter activity, they found that ISG20 inhibited translation for all exogenous DNA of both viral and host origins. However, when a CMV-GFP cassette was integrated into the host genome, ISG20 failed to inhibit GFP produced from this context as it did when the identical cassette was transfected into the same cells. They also generated a series of mutants outside of the invariant DEDD residues to explore how non-exonuclease regions impact ISG20 antiviral activity. Notably, mutations predicted to affect phosphorylation and cellular trafficking but not exonuclease activity still lose antiviral activity against VSV. This loss of antiviral activity correlates with inability to inhibit translation of non-self nucleic acids. Contrary to previous findings, the second group did not find that ISG20 induces IFIT1 expression in HEK293T or U937 cells. They propose instead that ISG20 recruits foreign RNA to P bodies, sites of RNA storage, where translation is repressed in the absence of RNA degradation (121).

All in all, ISG20 effectively inhibits viral replication by degrading or inhibiting translation of specific RNAs. ISG20 co-opts the cellular m^6^A pathway component, YTHDF2, to recognize and target modified HBV RNA (37). ISG co-factors such as IFIT1 may also be required in ISG20 translation inhibition, as ISG20 does not globally arrest translation like the OAS/RNase L pathway, but rather specifically targets non-self transcripts.

### ZAP

ZAP, encoded by the gene *ZC3HAV1*, is a potent antiviral factor which broadly inhibits replication of HIV-1 (*Retroviridae*), HBV (*Hepadnaviridae*), the (-) ssRNA viruses Ebola virus (EBOV, *Filoviridae*) and IAV (*Orthomyxoviridae*), and (+) ssRNA viruses such as alphaviruses (*Togaviridae*), coxsackievirus B3 (CVB3, *Picornaviridae*), and Japanese encephalitis virus (JEV, *Flaviviridae*) (125–132). ZAP antiviral activity can be selective within viral families and genera, as not all flaviviruses and picornaviruses tested are sensitive to ZAP (126, 131). It also post-transcriptionally regulates expression of cellular mRNA (133) and restricts retrotransposition of human retrotransposons (134, 135). ZAP possesses four N-terminal zinc fingers (136) that directly bind to viral RNA, are required for antiviral activity, and dictate its mostly cytoplasmic and stress granule localization (134, 137–139). Phosphorylation of this minimal antiviral N-terminal region by glycogen synthase kinase 3β enhances ZAP antiviral activity, though its mechanistic contribution remains unclear (140). Alternative splicing results in multiple splice variants which differ from one another in expression, localization, and antiviral activity. ZAP is also known as PARP13, due to poly-ADP-polymerase 13 (PARP13), due to the inclusion of a catalytically inactive C-terminal PARP-like domain in its long splice variant (ZAPL). In addition to a short splice variant that lacks this C-terminal domain (ZAPS), alternative splicing of a 121 aa extension of exon 4 results in two additional splice variants ZAPM and ZAPXL, whose antiviral activities are similar to ZAPS and ZAPL, respectively (141).

ZAP targets viruses primarily by two distinct antiviral mechanisms, namely viral translation inhibition (for the (+) ssRNA viruses SINV and JEV) and viral RNA degradation (for HIV-1, HBV, the (-) ssRNA virus EBOV, and the (+) ssRNA viruses CVB3 and JEV). These disparate mechanisms can be explained in part by recruitment of co-factors and differing viral contexts. ZAP inhibition of SINV translation has been linked to its disruption of the interaction between translation initiation factors eIF4A and eIF4G (142). This disruption does not affect global translation seeing as polysome profiles were unchanged when ZAP was overexpressed (142). ZAP is also able to repress translation of a luciferase reporter containing the minimal ZAP responsive fragment in the SINV genome without promoting degradation of the reporter (142). More recently, the E3 ligase tripartite motif-containing protein 25 (TRIM25) was uncovered as a novel ZAP co-factor in the context of alphavirus infection (47, 143). TRIM25 is absolutely required for inhibition of viral translation by ZAP, as ZAP is unable to inhibit translation of a replication-deficient reporter virus in TRIM25-deficient cells (47). Not only is TRIM25 putatively required for ZAP recognition of its RNA substrates, as TRIM25 knockdown decreases ZAP association with luciferase reporter RNA, but also TRIM25 ubiquitin ligase activity is essential for ZAP antiviral activity (47, 143). Curiously, though TRIM25 ubiquitinates ZAP, TRIM25 still contributes to ZAP antiviral activity in the presence of a ubiquitination-deficient ZAP mutant (47), suggesting that TRIM25-mediated ubiquitination of host factors other than ZAP is critical for the inhibitory effects (**Figure 4B**). Moreover, it is likely that K63-linked ubiquitination is required for ZAP antiviral activity, as overexpression of a ubiquitin K63R mutant unable to form K63 linkages reduces ZAP inhibition of SINV replication (143). The identity of these TRIM25 substrates that function in ZAP antiviral activity remains to be discovered, as does how they contribute to viral translation suppression.

Apart from inhibiting viral translation, ZAP also induces viral RNA degradation by recruiting an array of RNA helicases, the endonuclease KHNYN, and exosome components (144–147). ZAP selectively affects cellular transcripts, as it destabilizes the TRAILR4 mRNA and inhibits retrotransposition of endogenous retroelements such as Long INterspersed Element-1 (LINE-1) and Alu (133, 134). ZAP substrate specificity determinants largely remained a mystery until it was demonstrated to inhibit HIV-1 with synonymous, elevated CG dinucleotide mutations (HIV^CG^) but not wild-type HIV-1 (148). Interestingly, only elevation of CG dinucleotides in the 5’ third of the HIV-1 envelope gene caused ZAP susceptibility (149). Solving the crystal structure of ZAP in complex with CG-rich RNA revealed that ZAP has a CG-dinucleotide specific binding pocket and binds to ss nucleic acids (150, 151). ZAP preference for CG-rich substrates could explain in part why many RNA viruses infecting mammals and other vertebrates, such as IAV and SARS-CoV-2, exhibit CG suppression (152–155). ZAP can even sense CG dinucleotides within individual RNA transcripts of DNA viruses, as in the case of human cytomegalovirus (156). Here, CG suppression within the major immediate early transcript 1 confers ZAP resistance (156). However, CG suppression does not always confer resistance to ZAP as in the case of SARS-CoV-2 (154). Though initially identified in the context of translation inhibition, ZAP was also shown to form a complex with TRIM25 and the endonuclease KHNYN to inhibit HIV^CG^ (**Figure 4C**) and knockdown of KHNYN abolished HIV^CG^ sensitivity to ZAP (147).

All three ZAP antiviral activities of CG dinucleotide sensing, RNA degradation, and translation inhibition were linked in the context of JEV infection, wherein ZAP bound CG-rich regions of JEV RNA and inhibited translation at early time points without RNA degradation, and degraded RNA in an exosome-dependent manner at later time points of a JEV replicon (131). Therefore, ZAP can block viral translation in the context of alphavirus infection, target viral RNA for degradation in the context of retrovirus infection, and do both in the context of JEV infection.

To complicate matters further, ZAP splice variants also display differences in antiviral activity. ZAPL is more antiviral than ZAPS (141, 157). This boost to antiviral activity is attributed to its PARP-like domain. Not only does the PARP-like domain carry signatures of positive selection (157), but it also has a prenylation motif that targets ZAPL to endolysosomes (158, 159). Addition of this prenylation motif to ZAPS increases its antiviral activity, though not to the same extent as ZAPL (159). Curiously enough, ZAPL’s catalytically dead PARP triad motif is required for its antiviral activity; its replacement with the canonical active PARP motif abolishes ZAPL antiviral activity, though it remains unclear how this inactive motif is required (160). Furthermore, ZAPL is constitutively expressed in cells, while ZAPS expression is induced by innate immune signaling (141, 159). Studies conflict as to how ZAPS contributes to innate immune signaling. Though one group showed that ZAPS stimulates RIG-I dependent IFN response upon stimulation with a RIG-I RNA agonist (161), others found that ZAP mediates a RIG-I-independent antiviral response to retroviruses and HBV (128, 162, 163). More recently, ZAPS was shown to negatively regulate the type I IFN response by binding to and stimulating the degradation of *IFN* mRNAs; ZAP-deficient Huh7 cells had a higher and more prolonged IFN response upon treatment with a RIG-I agonist (159). On the other hand, ZAPS was found to synergize with other ISGs, wherein 31 ISGs have a statistically significant increase in antiviral activity in the presence of ZAP (32). In addition to its role as a co-factor in ZAP translation inhibition and CG sensing, TRIM25 may also modulate expression of ZAP isoforms by regulating alternative splicing, wherein TRIM25 is required for efficient expression of ZAPS (156).

All in all, ZAP inhibition of viral replication layers in complexity through its diverse mechanisms of translation inhibition and RNA degradation, recruitment of varied co-factors, and further differences between splice variants. The differing C-termini, expression kinetics, and cellular localization between splice variants could facilitate recruitment of divergent co-factors to enable different antiviral roles. As the PARP-like domain of ZAPL lacks any catalytic activity, it likely acts as an interaction domain to recruit specific co-factors that might be ADP-ribosylated to effect the RNA-centric antiviral mechanisms of ZAP.

### SHFL

SHFL, variously referred to as C19orf66, RyDEN, IRAV, or FLJ11286, is a 291 amino acid protein that is predicted to consist of eight α-helices and seven β-strands and possess both a nuclear export and localization signal, a zinc-ribbon domain, and a coiled-coil motif (164). SHFL binds nucleic acids and shows greater preference for ss nucleic acids and for RNA over DNA *via* fluorescence polarization assays (165). No catalytic activity is currently attributed to SHFL.

In uninfected cells, SHFL resides primarily in the cytoplasm in punctate structures, associating with both stress granule and P body proteins in HEK293 and Huh7.5 cells (165, 166), but was also more recently identified as an antiviral effector counteracting replication of RNA viruses (27, 28, 164, 167). For example, SHFL broadly inhibits replication of members of the (+) ssRNA virus family *Flaviviridae*, including all four DENV serotypes, WNV, ZIKV, and HCV (164–166, 168). SHFL also inhibits the virion production of chikungunya virus and SINV, members of the (+) ssRNA virus family *Togaviridae*. However, SHFL selectively inhibits members of another (+) ssRNA family, *Picornaviridae*, inhibiting replication of encephalomyocarditis virus but not poliovirus or enterovirus 71 (164, 165). SHFL selective inhibition also extends to DNA viruses, as its overexpression inhibits virion production of *Adenoviridae* member human adenovirus type 3, and *Herpesviridae* members KSHV and herpes simplex virus-1 (HSV-1), but not HSV-2 (164, 169).

A recently proposed mechanism for SHFL antiviral activity is suppression of viral translation. In line with this hypothesis, SHFL associates specifically with DENV RNA (164) and co-immunoprecipitates with other RNA-binding proteins that bind to mRNA 3’ UTRs such as PABPC1, LARP1, MOV10, and UPF1 (164, 165). Given that PABPC1 is critical for translation, overexpression of SHFL suppresses translation of a DENV replication-deficient luciferase reporter (164). Co-immunoprecipitation and immunofluorescence techniques were used to show that SHFL interacts with MOV10 and UPF1 even in the presence of RNase A, though the interaction was diminished (165). It is likely that SHFL mediates viral translation inhibition by interacting with the viral RNA 3’UTR binding proteins such as PABPC1 and LARP1 to block recruitment of further translation machinery (**Figure 4D**).

A separate, better characterized mechanism that SHFL utilizes to block viral translation is its broad inhibition of -1 programmed ribosomal frameshifting (-1PRF) which is crucial for many viruses to control protein expression levels (54). SHFL inhibits HIV-1 replication by altering the Gag to Gag-Pol protein ratio *via* inhibition of -1PRF, wherein knockdown of SHFL results in increased Gag-Pol expression without obviously altering either Gag or capsid expression (54). Noticeably, these data are in agreement with previous findings of unchanged p24 levels upon SHFL overexpression, which were originally interpreted as evidence that HIV-1 is not inhibited by SHFL (164). SHFL was also demonstrated to inhibit both viral and cellular mRNAs -1PRF signals in the context of a dual luciferase reporter construct (54). It is important to note that overall cellular translation and protein expression and ISG expression are unchanged in the presence of SHFL overexpression or knockdown in Huh7.5 cells (166), supporting the notion that SHFL antiviral activity is not due to modulating ISG expression or alteration of global translation (166).

However, SHFL may also act on some viruses independent of its direct effects on viral translation. For example, SHFL associates with the flavivirus replication complex in both DENV and ZIKV infections and interacts specifically with nonstructural protein 3 (NS3) in an RNA-independent manner (165, 168). By doing so, SHFL induces lysosomal-mediated degradation of NS3 in ZIKV-infected cells. SHFL is thought to inhibit HCV replication by interfering with the HCV-induced remodeling of the ER, which generates a membranous web that scaffolds assembly of viral replication complexes (166).

Taken together, SHFL inhibits viral replication by regulating translation through -1PRF-dependent and independent mechanisms and by specific antagonism of viral proteins and structures. SHFL displays both RNA-independent and -dependent interactions with other proteins, relying on RNA for its interaction with cellular RNA helicases MOV10 and UPF1 while interacting with flavivirus NS3 in the absence of RNA. These varied requirements for protein-protein interactions may reflect SHFL’s diverse antiviral mechanisms.

## Ring in Viral Transcription: Ubiquitin Ligase-Dependent and -Independent Inhibition

The really interesting new gene (RING) proteins are the most abundant family of E3 ligases, characterized by their N-terminal catalytic RING domain. E3 ligases occupy the final step in cellular ubiquitination. Ubiquitination of a protein can alter its cellular fate depending on the type of linkage, ranging from proteasomal degradation to scaffold formation for assembly of cellular signaling complexes (170). In order for ubiquitin to be ligated to an acceptor lysine, it must be sequentially activated by the E1 enzyme, carried by the E2 conjugating enzyme, and finally ligated to an acceptor lysine by one of over 600 human E3 ligases. Though many of the antiviral effectors mentioned in this section have other known cellular and antiviral roles, this section will focus on how IFN-inducible RING ligases inhibit viral transcription by both ligase-dependent and -independent mechanisms.

### RBBP6: Ligase-Independent Viral Mimicry

Retinoblastoma binding protein 6 (RBBP6) is a RING E3 ligase that inhibits transcription of the (-) ssRNA virus EBOV (*Filoviridae)* (53). RBBP6 was identified in an affinity-purification mass spectrometry screen to map host-EBOV protein-protein interactions as the most robust host interactor with the EBOV transcription regulator viral protein 30 (VP30) (53). RBBP6 competes with the EBOV nucleoprotein for binding to VP30 in an RNA-independent manner; the minimal RBBP6 interaction motif is sufficient to inhibit viral transcription, demonstrating a ligase-independent antiviral mechanism for RBBP6 (53). However, full-length RBBP6 causes dose-dependent decrease of VP30 protein in a manner dependent on the RBBP6-VP30 interaction, potentially suggesting a ligase-dependent antiviral mechanism (53). Curiously, RBBP6 also causes a dose-dependent decrease of VP30 mRNA independent of RBBP6-VP30 interaction. This suggests that RBBP6 either degrades VP30 mRNA through an uncharacterized exonuclease domain or recruits co-factor(s) that possess exonuclease activity (53). Knockdown of RBBP6 enhances EBOV RNA synthesis and replication. Taken together, these results suggest that RBBP6 inhibits EBOV replication through a three-pronged approach, and that both ligase-dependent and -independent antiviral mechanisms and exonuclease-dependent mechanism may be waiting to be further characterized.

### TRIMming Viral Transcription

The tripartite motif containing proteins (TRIM) are the largest group of RING E3 ligases and constitute an important family of proteins in the type I IFN response (171, 172). There are over 70 human TRIM proteins, many of which are induced by type I IFN (171, 173). Interestingly, the rapid expansion of the TRIM family coincides with the development of adaptive immunity, suggesting that TRIMs may have evolved to play a role in immune regulation (171). These proteins typically possess three conserved domains at the N-terminus—a catalytic RING domain, one to two B-box domains that are thought to function in higher order oligomerization, and a coiled-coil domain that allows TRIMs to dimerize and potentially oligomerize (174). Most TRIMs directly inhibit viral replication by targeting viral components for degradation, or indirectly inhibit by modulating innate immune signaling (175, 176).

Multiple TRIM members have been found to inhibit viral transcription *via* both ligase-dependent and -independent mechanisms. TRIM22 does both, though its ligase activity is required to inhibit HBV transcription, it inhibits HIV-1 transcription independent of its ligase activity (177–179). TRIM22 inhibits HBV core promoter activity, which is critical for HBV pgRNA synthesis and hence viral transcription and reverse transcription (177, 180). A single point mutation in its RING domain abolishes its inhibition of viral replication, strongly implicating ligase activity in anti-HBV effects of TRIM22 (177). Meanwhile, TRIM22 inhibits HIV-1 basal transcription independent of its ligase activity by indirectly preventing the transcription factor specific protein 1 (Sp1) from binding to the HIV-1 promoter, thus facilitating HIV-1 latency (181, 182). As TRIM22 neither directly interacts with Sp1 nor binds to the HIV-1 promoter, it is possible that TRIM22 recruits another co-factor to alter chromatin state or stimulate Sp1 post-translational modification (181). Two independent TRIMs inhibit IAV transcription, a (-) ssRNA virus. TRIM32 depends on its ligase activity, ubiquitinating the core component of the IAV RNA polymerase complex and targeting it for degradation, subsequently reducing polymerase activity (57). On the other hand, TRIM25 restricts IAV RNA synthesis in a ligase-independent manner by binding to viral ribonucleoproteins and blocking RNA chain elongation (56).

TRIM69 is a more recently identified ISG and antiviral effector that shares high homology with TRIM25. Recently, two independent groups found that TRIM69 inhibits replication of the Indiana strain of VSV (VSV_IND_), a (-) ssRNA virus, in ligase-independent manner (58, 59). Both groups identified TRIM69 through targeted screens using complementary approaches, either overexpressing an array of known ISGs or knocking down VSV-induced host genes (58). In addition, both groups found that TRIM69 inhibition of VSV is highly specific, as overexpression of TRIM69 fails to inhibit the New Jersey strain of VSV (VSV_NJ_) or other negative-strand RNA viruses such as SeV, rabies virus, or IAV (58, 59). VSV_IND_ sensitivity to TRIM69 was mapped to a short peptide sequence within the viral phosphoprotein P by serial passaging VSV_IND_ in the presence of TRIM69 overexpression and sequencing escape mutants (58, 59). VSV_NJ_ differs from VSV_IND_ at five out of six amino acids within this TRIM69 P sensitivity determinant, potentially explaining differential resistance between VSV strains. TRIM69 physically associates with VSV_IND_ P but does not require its ligase activity or target it for degradation. Instead, TRIM69-inhibition of VSV_IND_ requires its multimerization in order to sequester VSV_IND_ P into filamentous structures, thus disrupting viral replication machinery.

To summarize, RING E3 ligases combat viral transcription in myriad ways. Many do not rely on their ligase activity to inhibit transcription by directly binding to components of viral transcription machinery to inhibit protein-protein interactions, such as RBBP6, TRIM25, and TRIM69. Others ubiquitinate viral components to target them for degradation, such as TRIM22 and TRIM32.

## Discussion

Inhibition of viral replication by ISGs grows more nuanced as every new study promises to uncover new facets of antiviral or pro-viral activity. Even well-characterized ISGs such as OAS/RNase L, ISG20, and ZAP have had new aspects of their antiviral mechanisms come to light in recent years. RNA viruses present a plethora of unique viral RNA processes that host cells can identify and inhibit. They rely on their own viral RNA-dependent RNA polymerases to transcribe and replicate genomic RNA, generating dsRNA intermediates that host cells recognize as foreign. Each RNA-centric antiviral mechanism mentioned in this review affords specific advantages and disadvantages. Blocking viral translation is especially effective against positive-stranded RNA viruses, which must translate their incoming genomes before any further steps in viral replication can occur. Likewise, inhibiting viral transcription is especially effective against negative-stranded RNA viruses, which prioritize transcribing their genomes upon entry. While degrading genomes outright appears to be the most straightforward and universal way to inhibit replication of RNA viruses, RNA degradation presents its own set of challenges of distinguishing foreign from self RNA. In the case of OAS/RNase L, dsRNA sensing by OAS leads to virtually indiscriminate degradation of viral and host RNAs, curtailing viral replication but also killing the infected cell. Some especially pathogenic viruses such as Middle East respiratory syndrome coronavirus (MERS-CoV) circumvent RNA degradation by enzymatically degrading 2-5A, thus preventing RNase L activation (183). Meanwhile, other ISGs, such as ZAP, recognize specific motifs in the viral genome distinct from host genomes so only viral RNA is selectively targeted. However, ZAP specificity for CG dinucleotides may have driven selection against high CG content in RNA viral genomes, thus potentially rendering ZAP ineffective (152, 153).

The perpetual arms race between antiviral effectors and viruses has likely driven the development of multilayered mechanisms of viral inhibition. Some individual ISGs have acquired multiple antiviral mechanisms, enabling them to circumvent viral evasion. For example, ZAP is still able to inhibit replication of SARS-CoV-2, a positive-sense ssRNA virus with highly suppressed CG content (154). This suggests that ZAP inhibits SARS-CoV-2 not by CG sensing alone and that there are likely additional sequence or structural motifs that are recognized and targeted by ZAP. Another way ISGs may prevent viral evasion is through the use of co-factors, which could function as a natural “antiviral cocktail” (**Figure 4**). By using co-factors, host cells employ a multipronged attack on viral replication that could help minimize evasion by RNA viruses. ISGs that work in concert to recognize specific viral RNA motifs could make it difficult for viruses to simultaneously mutate all recognizable motifs in their genomes. Though it is known that TRIM25 functions as a ZAP co-factor (47, 143), that both TRIM25 and ZAP bind RNA (48, 139, 184, 185), and that RNA binding is crucial for their antiviral activity (136, 185), it remains unexplored whether TRIM25 and ZAP work together to recognize viral RNA substrates or motifs or both. TRIM25 and ZAP putative cooperation in viral recognition could help explain why many alphaviruses have not acquired ZAP resistance and remain acutely sensitive to its inhibition (126, 141).

In-depth characterization of ISG antiviral mechanisms and methods of viral evasion has been facilitated by rapid expansion of CRISPR-Cas technologies. For example, identification of OAS3 as necessary and sufficient for RNase L activation was enabled by generation of single OAS KO cell lines (67). CRISPR-Cas technologies have also enabled the discovery and interrogation of functions of novel ISGs and their splice variants or polymorphisms (186). Genome-wide CRISPR-Cas9 KO and targeted ISG overexpression screens have been used to great effect, identifying novel host factors and highlighting important antiviral ISGs to characterize [reviewed in (20)]. One exciting new avenue for RNA-centric ISG identification lies at the intersection of chemical biology. Generation of nucleotide analogs that can be incorporated into RNA, cross-linked to proximally bound proteins, and immunoprecipitated for subsequent proteomic analysis enables the identification of novel RNA-binding proteins (187). Variations on this approach have been used several times in the context of positive-sense RNA virus infections by *Togaviridae* and *Flaviviridae* family members to elucidate new host-virus interactions (49, 188–190). It is not always feasible to target host factors required for viral replication, since these host factors may also be essential for cell survival. Furthermore, overexpression of any given protein may yield false phenotypes that are not biologically relevant. In contrast, *in situ* labeling and identification of endogenous RNA-binding proteins offers a more specific and minimally disruptive approach with fewer effects on cell viability. Cross-referencing ISGs with novel viral RNA-binding proteins could yield promising candidates to characterize and open up new horizons for antiviral exploration.

## Author Contributions

EY and ML determined the scope and focus of the review. EY drafted the manuscript and generated all figures. ML provided critical feedback on the manuscript. All authors contributed to the article and approved the submitted version.

## Funding

This work was supported in part by the UC Cancer Research Coordinating Committee Faculty Seed Grant (CRN-20-637544; M.M.L.), UCLA AIDS Institute and Charity Treks 2019 Seed Grant (ML), and Ruth L. Kirschstein Multidisciplinary Training Grant in Microbial Pathogenesis (NRSA AI007323; EY).

## Conflict of Interest

The authors declare that the research was conducted in the absence of any commercial or financial relationships that could be construed as a potential conflict of interest.
